# Efficacy of insecticides used in indoor residual spraying for malaria control: an experimental trial on various surfaces in a “test house”

**DOI:** 10.1186/s12936-019-2969-6

**Published:** 2019-10-10

**Authors:** Ana Paula S. A. Corrêa, Allan K. R. Galardo, Luana A. Lima, Daniel C. P. Câmara, Josiane N. Müller, Jéssica Fernanda S. Barroso, Oscar M. M. Lapouble, Cynara M. Rodovalho, Kaio Augusto N. Ribeiro, José Bento P. Lima

**Affiliations:** 1 Laboratório de Fisiologia e Controle de Artrópodes Vetores–Fundação Oswaldo Cruz, Rio de Janeiro, Brazil; 2 Laboratório de Entomologia Médica, Instituto de Pesquisas Científicas e Tecnológicas do Estado de Amapá-IEPA, Macapá, Brazil; 3 Núcleo Operacional Sentinela de Mosquitos Vetores - Laboratório de Mosquitos Transmissores de Hematozoários, Fundação Oswaldo Cruz, Rio de Janeiro, Brazil; 4 Pan-American Health Organization/World Health Organization (PAHO/WHO), Paramaribo, Suriname; 5 Fundação de Medicina Tropical Dr. Heitor Vieira Dourado, Manaus, Amazonas Brazil; 6 Programa de Pós-Graduação em Medicina Tropical, Universidade do Estado do Amazonas, Manaus, Amazonas Brazil; 7 Santo Antônio Energia S/A, São Paulo, Brazil

**Keywords:** *Anopheles*, Wall surface type, Indoor residual spraying, Malaria, Integrated vector management

## Abstract

**Background:**

Malaria is a public health problem in the Brazilian Amazon region. In integrated vector management for malaria (anopheline) control, indoor residual spraying (IRS) represents one of the main tools in the basic strategy applied in the Amazonian states. It is essential to understand the residual efficacy of insecticides on different surfaces to determine spray cycles, ensure their rational use, and prevent wastage. This study aimed to evaluate the residual efficacy of six insecticide formulations used in the National Malaria Control Programme on four different types of walls in a field simulation at a “test house”.

**Methods:**

The tests were performed as a field-simulating evaluation at a “test house” built in the municipality of Macapá. Six insecticide formulations comprising four pyrethroids, a carbamate, and an organophosphate were used, and evaluated when applied on different wall surfaces: painted wood, unpainted wood, plastered cement, and unplastered cement. The insecticides were applied to the interior walls of the “test house” by a trained technician.

**Results:**

In the bioassays performed with pyrethroids, deltamethrin water-dispersible granules (WG) performed particularly well, presenting residual bioefficacy of 8 months on both wood surfaces after the IRS, whereas alpha-cypermethrin suspension concentrate (SC) and etofenprox wettable powder (WP) demonstrated residual bioefficacy of 4 months on at least one of the wood surfaces; however, the pyrethroid lambda-cyhalothrin WP showed a low residual bioefficacy (< 3 months) on all tested surfaces, demonstrating its inefficiency for areas with a long transmission cycle of malaria. For the carbamate-bendiocarb WP, residual bioefficacy for 3 months was achieved only on wood surfaces. In general, the organophosphate pirimifos-methyl capsule suspension (CS) demonstrated the best result, with a mortality rate < 80% over a period of 6 months on all surfaces tested.

**Conclusion:**

Insecticide efficiency varies among different types of surface; therefore, a “test house” is a valuable evaluation tool. This work highlights the usefulness of associating the residual efficacy of insecticides on the surfaces commonly found in houses in endemic areas, together with knowledge about the transmission cycle duration of the transmission cycle and the insecticide susceptibility of the vector. This association helps in the decision-making for the malaria control intervention regarding.

## Background

Malaria is an avoidable and treatable disease, but it remains one of the most serious public health problems globally. In endemic countries, poor, disadvantaged people with limited access to healthcare facilities are the most affected [1]. Approximately 90% of malaria cases in the Americas are reported in the Amazonian parts of South America, Bolivia, Brazil, Colombia, Ecuador, French Guiana, Guyana, Peru, Suriname, and Venezuela, with cases being mainly concentrated in Venezuela and Brazil [2]. According to the Brazilian Malaria Epidemiological Surveillance Information System (SIVEP/Malaria) [3], 99.8% of the cases are reported in the Amazon region, considered the endemic area, with high rates in states such as Amazonas, Acre, Pará, and Amapá [4, 5].

The set of interventions recommended by the World Health Organization (WHO), and adopted by the National Malaria Control Programme (NMCP), proposes: reducing the lethality and severity of cases, reducing the incidence of the disease through the elimination of transmission in urban areas, and maintaining the absence of the disease in places where the transmission has already been interrupted. This approach is understood to involve integrated, selective, and economic control activities that are suitable for the epidemiological scenario and appropriate to the actual conditions in each region [1, 6]. Among the proposed activities, vector control is an essential component and should be implemented based on local entomo-epidemiological data; for this, long-lasting insecticidal nets (LLIN) and indoor residual sprays (IRS) can be widely applied, which have achieved decreases in malaria cases [7, 8] in Africa, Asia, Europe, and Latin America [9–11].

Some limitations in *Anopheles* neotropical bioassays include a lack of mosquito colonization in laboratory circumstances and low availability of bioassays, except for colonies of *Anopheles aquasalis* and *Anopheles albitarsis* sensu stricto, which were kept in a laboratory by the Oswaldo Cruz Foundation in Rio de Janeiro, Brazil [12, 13]. Therefore, it is a priority to establish colonies of vectors that transmit malaria in Brazil [14]. Although malaria represents a serious public health problem in Brazil, few studies have evaluated the resistance of vectors to insecticides [15, 16]. However, Silva et al. [17] evaluated the susceptibility profile of insects to pyrethroids in the larvae of *Anopheles darlingi* and *Anopheles marajoara*, using a simple, fast, and low-cost methodology, as an alternative to traditional, certified tests in the Amazonian states of Brazil, with the results showing susceptibility in the populations in the municipality of Macapá.

The development of insecticides that remain active for long periods was one of the most important advances in insect control in the 20th century. Controlling malaria with insecticides in the Amazon dates back to the 1940s when two-thirds of the Brazilian population lived in endemic areas. Thus, a national campaign for the eradication of the disease with the expressive use of organochlorine DDT (dichlorodiphenyl-trichloroethane) in an organized and systemic manner [18, 19] was initiated, also contributing to the prevention of the epidemic of typhus transmitted by lice [20].

Organophosphates (e.g., malathion, temephos, and fenitrothion.) were developed in the 1940s and have been used ever since as insecticides, herbicides, and plant growth regulators. In the 1970s, organochlorines were replaced by organophosphates [21]; although they are biodegradable and non-cumulative, they present chemical instability and high toxicity in vertebrates [22]. Carbamates present a rapid lethal action; however, as well as organophosphates, their persistence in the environment is short, making more frequent applications necessary [23]. The adoption of pyrethroids in the fight against malaria vectors in Brazil began in the mid-1990s, with cypermethrin in a wettable powder formulation [16], with lambda-cyhalothrin 5% for thermonebulization, and etofenprox WP 20% for BRI [24]. In addition, LLIN is impregnated with pyrethroids. However, the judicious use of chemical insecticides is necessary, avoiding the contamination of the environment and the selection of populations of resistant vectors [25, 26].

Knowledge about the persistence of insecticides (residual effect) is essential to determine the appropriate frequency of insecticide application in dwellings in areas with a high malaria incidence, considering the duration of the malaria transmission season, and to systematize the cycles of application of such products [16, 27–29]. This activity consists of spraying the walls of residences with insecticides that remain in place on the applied surfaces. The residual efficacy of this is evaluated by performing bioassays as recommended by the WHO [30, 31], which should ideally be conducted in actual human habitations. However, factors such as the operational cost of mobilizing teams to perform this, the difficulty in accessing areas with houses sprayed with insecticides, ethical considerations [32], the variability of dwellings, and the non-use of F_1_ generation mosquitoes of a known age compromise the performance of such tests in field conditions [33]. On the other hand, laboratory panel bioassays, under controlled conditions, have demonstrated a more prolonged residual effect [34], which may lead to a longer interval in the spray cycles.

Historically, the use of experimental huts began in the 1940s, with the goal of capturing mosquitoes. Subsequent works adapted the models for studies including the evaluation of IRS and LLIN, repellents, and synthetic attractants [35–38]; such dwellings may even be transportable, such as the modified experimental Ifakara hut [39], which also exhibited the proven merits of the previously developed huts; however, there are no studies of studies of this nature in the Brazilian Amazon region, despite this being the location of the majority of the cases reported in the country [2]. The use of a “test house” for the field simulation for the wall bioassays can minimize the differences between the laboratory and the field; it can also decrease the operational costs, providing a better solution to define the spray cycles for IRS.

The aim of the present study was to evaluate, for the first time in a field simulation, the residual effect of six insecticide formulations used by the NMCP in an experimental hut called a “test house”, featuring walls composed of four different surfaces in the Brazilian Amazon.

## Methods

### Study area

**Fig. 1:**
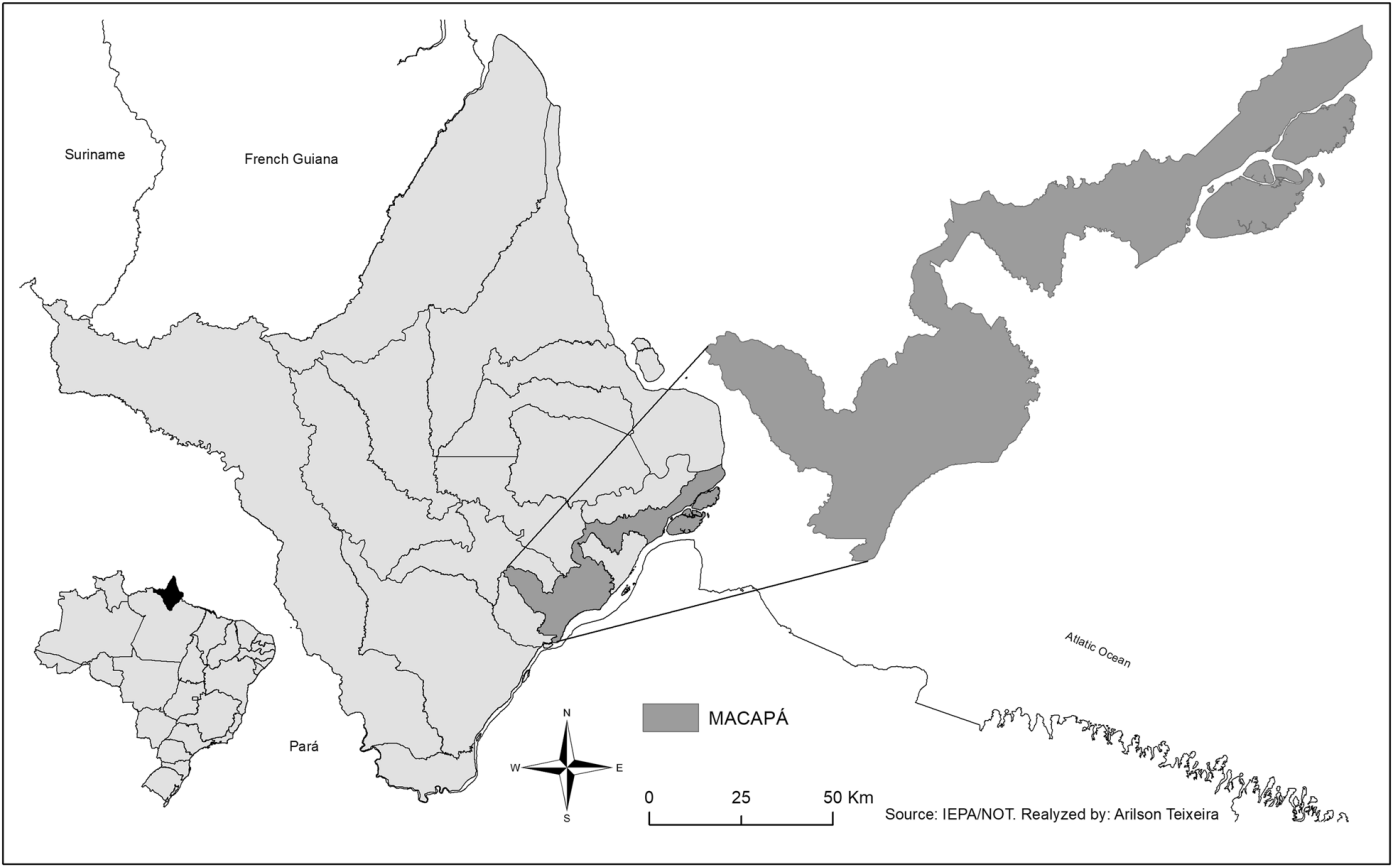
Spatial representation of the study area: Macapá—Amapá, Brazil

This study was carried out in the city of Macapá (latitude: 0°2′20″N, longitude: 51°3′59″W), in the state of Amapá (Fig. 1). This site has a humid equatorial climate with an annual average temperature of 27 °C and two well-defined seasons: summer (drought period, from July to December) and winter (rainy season, from January to June) [40]. Malaria is endemic in this region, with its incidence peaking in the period from August to November SIVEP-Malaria (http://200.214.130.44/sivep_malaria/).

### Study period

The study was conducted from October 2014 to March 2016, in the external area of the Laboratory of Medical Entomology-Campus Fazendinha, Institute of Scientific and Technological Research of the State of Amapá-IEPA, in two phases:

#### First phase

October 2014 to April 2015—testing of the insecticides alpha-cypermethrin SC, etofenprox WP, and lambda-cyhalothrin WP.

#### Second phase

May 2015 to March 2016—testing of the insecticides deltamethrin WG, bendiocarb WP, and pirimiphos-methyl CS.

### “Test house”

**Fig. 2:**
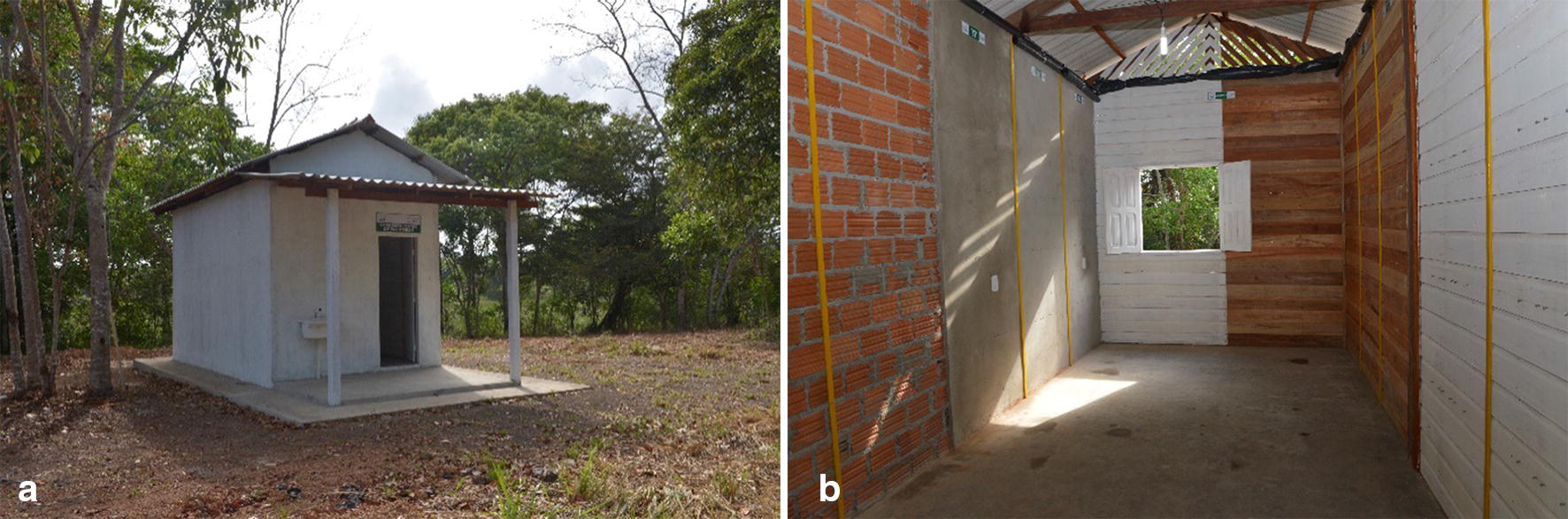
Test house external view. **a** Frontal and lateral view of the external plastered cement surfaces painted with acrylic paint. **b** The view of the side surfaces of unplastered cement (CP2) and plastered cement (CP1) divided into strips for the application of insecticides; view of the back wall (control) consisting of a painted wooden wall (WP1) and an unpainted wooden wall (WP2); a corrugated fiber cement roof and a bare concrete floor

A house was built in the external area of the Laboratory of Medical Entomology (IEPA) with the following characteristics: internal dimensions (3 m wide, 6 m long, 3 m high), a corrugated fiber cement roof supported by hardwood beams, unlined, and a bare concrete floor with a window measuring 1 × 0.74 m and a wooden door measuring 0.85 × 2.10 m. Externally, it had a pavement which was 0.70 m wide surrounding the house and a front porch of 2 m with the characteristics of the floor and cover (Fig. 2).

**Fig. 3:**
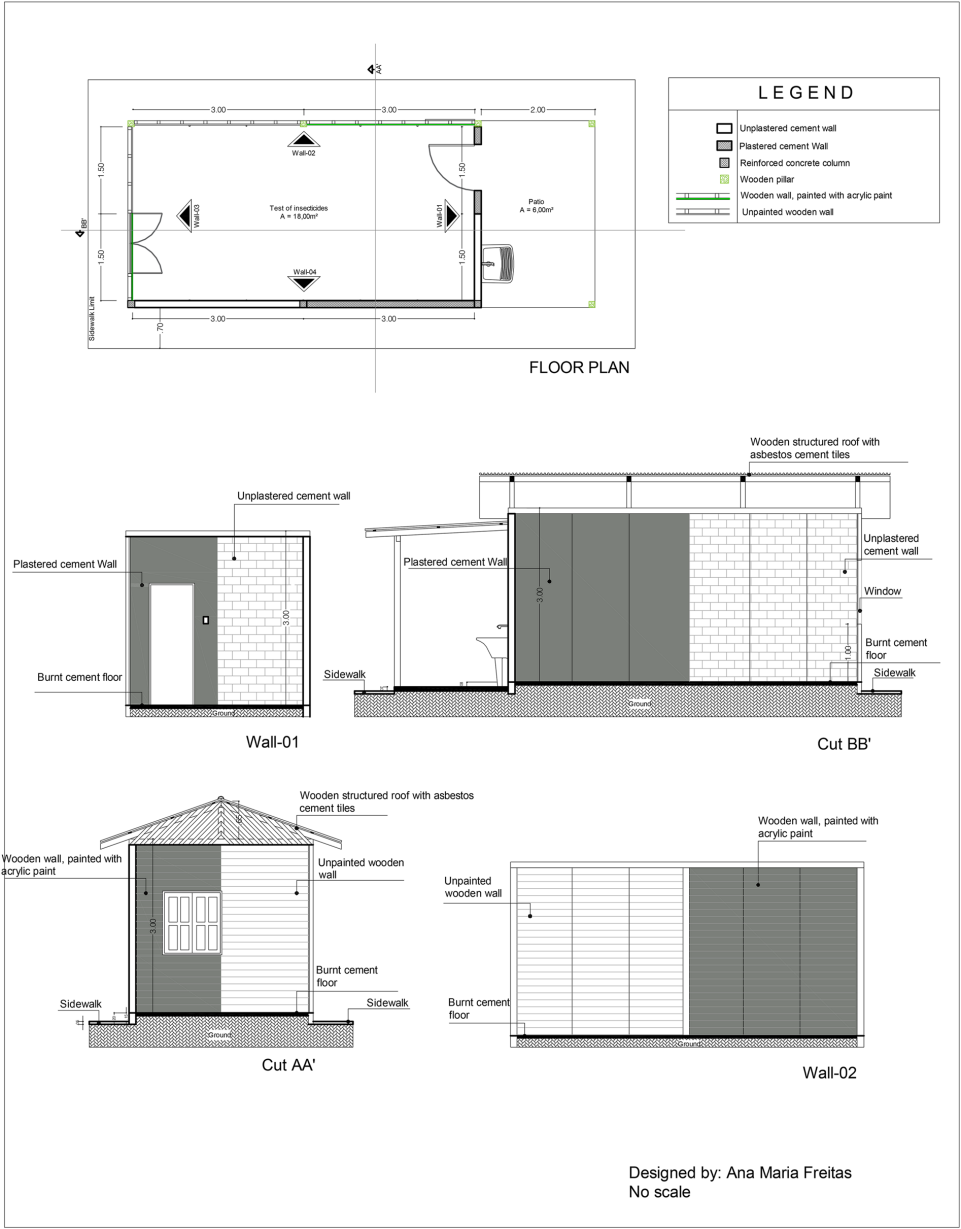
Representations of the “test house” built in Macapá-Amapá. This figure shows the floor plan (**a**), frontal and side plans of the plastered cement (CP1) and unplastered cement (CP2) surfaces (**b**), and back and side plans of the painted wooden (WP1) and unpainted wooden (WP2) surfaces, highlighting the important characteristics

The front wall and one of the side walls were made of bricks (masonry), whereas the other side wall and the back of the house were made of wood. Each side wall was divided into two parts. For the masonry wall, one part was left with only bricks, whereas the other was covered with plastered cement, referred to as plastered cement (CP1) and unplastered cement (CP2) surfaces. For the wooden wall, one part was painted with white acrylic paint, and the other part was kept without any paint, which is referred to as painted wood (WP1) and unpainted wood (WP2) surfaces. The front masonry wall was divided into two parts, CP1 and CP2, constituting the surfaces used as the controls. The same procedure was performed for the wooden back wall, where the WP1 and WP2 surfaces were used as the controls. Each side wall was divided into three 1-m-wide strips, and each strip was sprayed with one insecticide (Fig. 3).

The choice of the surfaces for the walls of the “test house” was based on the typical characteristics of the houses in the Amazon region. Houses in this region are also typically built on stilts. However, considering that the areas in which malaria is endemic are generally rural (e.g., settlements, villages, and districts) or forested, housing at these sites commonly features construction materials such as wood (abundant in the region), bricks, and cement, along with sand and straw roofs, ceramic tiles, or cement.

### Insecticides

Six insecticide formulations were evaluated, with the first analysis focusing on three pyrethroids, followed by a second analysis on a pyrethroid, a carbamate, and an organophosphate. The chemicals were used at the maximum concentrations for each formulation according to the World Health Organization Pesticide Evaluation Scheme (WHOPES) recommendations [41, 42], and included: (1) alpha-cypermethrin—ALFATEK^®^ 200 SC, sprayed at a concentration of 0.03 g a.i./m^2^ (grams of active ingredient), lambda-cyhalothrin—ICON^®^ 10 WP sprayed at a concentration of 0.03 g a.i./m^2^, and etofenprox—VECTRON^®^ 20 WP, sprayed at 0.3 g a.i./m^2^; (2) deltamethrin—DELTAGARD^®^ 250 WG sprayed at a concentration of 0.025 g/m^2^, bendiocarb—FICAM^®^ VC—WP at 0.4 g a.i./m^2^, and pirimiphos-methyl CS—Experimental Sample (Syngenta, Switzerland) sprayed at 1 g a.i./m^2^. The compounds have complete or provisional WHO approval and represent a diverse range of common insecticides currently used in vector control.

### Wall bioassays—residual efficacy tests

Considering that most species of Brazilian anophelines are not yet colonizable in the laboratory, obtaining a sufficient number of individuals to be used in the bioassays is difficult. *Anopheles* (Nyssorhynchus) *marajoara*, was chosen for the residual efficacy bioassays because of the high population density of the vector that can be captured abundantly using animals as attractive. These anophelines are collected directly from the walls of buffalo corrals with the help of the mouth aspirator in rural areas of the region. In addition, *An. marajoara* is a vector of the complex Albitarsis, which has been implicated as the main vector in some municipalities of the state of Amapá [43–45], with anthropophilic and zoophilic, endo and exophageal behaviour, but being almost exclusively exophilic [46].

Against this background and considering the work of Silva [47] on the susceptibility/resistance of this vector in the Amazon region, including the state of Amapá, it was considered that populations collected in areas with little or no use of insecticides could be used for conducting bioassays. As such, the population of *An. marajoara* was used for this study since the samples were collected monthly, in the municipality of Mazagão, with the aid of a mouth aspirator at sites in which insecticides have not been directly applied. Females of *An. marajoara* (F_1_ generation) were raised in the Insectary Laboratory of Medical Entomology/IEPA in accordance with a modified version of the protocol of Horosko et al. [12].

For the two phases in this study, with the exception of the control strips, the water-diluted insecticides were applied to the inner walls of the “test house” with a Hudson X-Pert nozzle type 8002-E pump by a trained technician from the Amapá State Secretary of Health. This was supervised by IEPA technicians and performed in accordance with the WHO guidelines, with the following specifications: a pressure of 25–55 psi, the distance from the tip of the nozzle to the sprayed surface of 45 cm, and a sprayed strip width of 75 cm [7, 48, 49].

To avoid cross-contamination between the insecticides at the time of spraying, they were applied on different days of the same week. Additionally, all the internal walls were completely sealed with a waterproof plastic tarpaulin, being exposed only four different swaths per surface type (CP1, CP2, WP1, and WP2), which were then sprayed with the specific insecticide. This tarpaulin was only removed after the insecticide had completely dried.

In the bioassays, each strip of the tested surface received nine plastic cones, distributed at three heights corresponding to 0.5, 1.0, and 1.5 m above the ground. For the control walls, one cone was used for each height. All the cones received approximately 15 mosquitoes [30, 31, 50–53]. After 30 min of exposure to the treated walls, the mosquitoes were transferred from the cones to clean entomological cups, where the first reading was performed. Subsequently, the mosquitoes were taken to the laboratory located on the same campus as the “test house”, fed 10% sucrose solution and, stored in a humid chamber, with a temperature between 25 and 27 °C and relative humidity between 70 and 80%.

The mortality rate was calculated 24 h after the end of the test, considering live mosquitoes as those capable of flying after slight agitation in the entomological cup, regardless of the degree of damage suffered. Abbott’s formula was used to correct the mortality rates when there was a mortality rate of between 5 and 20% in the control group. No correction was necessary when the mortality rate was below 5%, while the bioassay was repeated when the rates were above 20% [54].

The first bioassay was carried out 1 day after the application of the insecticides, and then at approximately 30-day intervals. The mortality rates in the exposed group were established as satisfactory when they were greater than or equal to 80%, in accordance with the WHO criteria [30]. In this study, the end of the evaluation period for each insecticide was defined as when a reduction in the mortality rate occurred for two consecutive months or for up to 240 days after surface spraying for insecticides that maintained a mortality < 80%.

In the interval from one stage to the next, the test house was cleaned, and its walls were washed several times with the aid of neutral soap and a high-pressure washer for the complete removal of the residues from the applied insecticides. After each wash, the cleaning of the wall was verified with wall bioassays, following the recommended methodology with an expected 0% mortality rate. Once this rate had been observed on all the internal surfaces of the test house, it was released for the application of the insecticides in the second stage.

### Climate data

Environmental data concerning relative humidity, temperature and rainfall were obtained at the Fazendinha campus meteorological station of the Hydrometeorology and Renewable Energies Nucleus/IEPA.

### Data analysis

Statistical analyses were performed using R [55] and RStudio [56] with a significance level of 5%. A series of logistic regression models were used to estimate the residual effect of six different formulations of insecticides on the mortality of *An. marajoara* exposed on four types of surfaces up to 8 months after the initial application of the insecticide. Similar data analysis was used to estimate the effects of the surface type and cone height on *An. marajoara* mortality. A separate model was established for each of the six formulations of insecticides used: alpha-cypermethrin SC, etofenprox WP, lambda-cyhalothrin WP, deltamethrin WG, bendiocarb WP, and pirimiphos-methyl CS. The models tested the surface type 1 month after the initial exposure and its interaction with fixed effects. The mortality was calculated after 24 h of product exposure. When significant effects were found, follow-up analyses were performed for paired comparisons using the Bonferroni method available in the lsmeans package [57].

Average temperature and mean relative humidity were compared in both experimental phases using a Welch’s *t* test, due to unequal sample size (187 days in phase 1 and 281 days in phase 2). The total number of rainy days was compared in each phase using a Wilcoxon–Mann–Whitney test.

The exclusion criterion for the statistical analyses was a mortality rate that did not meet the manufacturers’ specifications of a residual effect varying from 2 to 6 months. In this way, insecticides with a mortality rate below 80% in a period shorter than 60 days were excluded from the tests.

## Results

The results presented in the tables and line graphs demonstrate the decay rate of the six different insecticides expressed as the 24 h mortality rate *versus* the number of days post spraying on the four different surfaces. Here the survival of the mosquitoes is considered as an indicator of the decreased of the residual effect of the insecticide on a given surface. The evaluation of the residual effect of the insecticides was carried out considering the application of different formulations on distinct surfaces: WP1, WP2, CP1, and CP2 (Tables 1, 2).

**Table 1:** The mean (SE) monthly mortality rate of *An. marajoara* after 24 h post-exposure to six different insecticides sprayed on four different surfaces: painted wooden wall (WP1), unpainted wooden wall (WP2), plastered cement wall (CP1), and the unplastered cement wall (CP2)

Insecticide	Substrate	Days post IRS								
		1	30	60	90	120	150	180	210	240^a^
Alpha-cypermethrin SC	WP1	1.00 (−)	1.00 (−)	0.99 (0.02)	0.78 (0.04)	0.81 (0.04)	0.49 (0.07)	0.25 (0.06)	–	–
	WP2	0.82 (0.05)	0.92 (0.03)	0.47 (0.14)	0.43 (0.12)	–	–	–	–	–
	CP1	0.99 (0.02)	0.77 (0.06)	0.22 (0.05)	–	–	–	–	–	–
	CP2	0.97 (0.03)	0.51 (0.04)	0.49 (0.08)	–	–	–	–	–	–
Etofenprox WP	WP1	1.00 (−)	1.00 (−)	0.85 (0.04)	0.82 (0.05)	0.58 (0.10)	0.30 (0.08)	–	–	–
	WP2	1.00 (−)	0.97 (0.02)	0.98 (0.03)	0.97 (0.02)	0.86 (0.06)	0.68 (0.08)	0.33 (0.08)	–	–
	CP1	1.00 (−)	0.93 (0.03)	0.87 (0.05)	0.40 (0.07)	0.54 (0.08)	–	–	–	–
	CP2	0.97 (0.02)	0.67 (0.11)	0.54 (0.07)	–	–	–	–	–	–
Lambda-cyhalothrin WP	WP1	1.00 (−)	0.98 (0.02)	0.62 (0.11)	0.43 (0.07)	–	–	–	–	–
	WP2	0.96 (0.02)	0.96 (0.03)	0.73 (0.05)	0.63 (0.10)	–	–	–	–	–
	CP1	0.88 (0.04)	0.79 (0.06)	0.03 (0.02)	–	–	–	–	–	–
	CP2	0.98 (0.02)	0.74 (0.05)	0.60 (0.11)	–	–	–	–	–	–
Deltamethrin WG	WP1	1.00 (−)	1.00	0.98 (0.03)	0.97 (0.03)	0.95 (0.02)	0.94 (0.02)	0.85 (0.05)	0.82 (0.04)	0.83 (0.06)
	WP2	1.00 (−)	0.98 (0.02)	0.58 (0.08)	0.94 (0.03)	0.94 (0.03)	0.94 (0.03)	0.92 (0.04)	0.98 (0.02)	1.00 (−)
	CP1	1.00 (−)	1.00 (−)	0.90 (0.06)	0.87 (0.04)	0.74 (0.07)	0.68 (0.08)	–	–	–
	CP2	1.00 (−)	0.99 (0.02)	0.85 (0.06)	0.73 (0.13)	0.66 (0.07)	–	–	–	–
Bendiocarb WP	WP1	1.00 (−)	1.00 (−)	1.00 (−)	1.00 (−)	0.53 (0.13)	0.60 (0.12)	–	–	–
	WP2	1.00 (−)	1.00 (−)	1.00 (−)	1.00 (−)	0.46 (0.12)	0.65 (0.14)	–	–	–
	CP1	1.00 (−)	0.11 (0.03)	0.03 (0.02)	–	–	–	–	–	–
	CP2	1.00 (−)	0.05 (0.03)	0.06 (0.02)	–	–	–	–	–	–
Pirimiphos-methyl CS	WP1	1.00 (−)	1.00 (−)	1.00 (−)	0.98 (0.03)	1.00 (−)	1.00 (−)	1.00 (−)	0.98 (0.02)	0.79 (0.10)
	WP2	1.00 (−)	1.00 (−)	0.94 (0.04)	0.79 (0.08)	0.84 (0.05)	0.87 (0.05)	0.73 (0.10)	0.66 (0.07)	–
	CP1	1.00 (−)	0.98 (0.03)	1.00 (−)	0.92 (0.06)	0.69 (0.11)	0.95 (0.04)	0.65 (0.09)	1.00 (−)	0.90 (0.06)
	CP2	1.00 (−)	0.97 (0.02)	1.00 (−)	0.98 (0.02)	1.00 (−)	0.98 (0.02)	0.97 (0.04)	0.92 (0.03)	0.98 (0.03)

^a^The experiments were maintained until a mortality rate of less than 80% was observed for two consecutive months

**Table 2:** The odds ratio [95% confidence interval] of monthly mortality rates of *An. marajoara* after 24 h post-exposure to alpha-cypermethrin, etofenprox WP, and lambda-cyhalothrin CS on four different surfaces: plastered cement wall (CP1), unplastered cement wall (CP2), painted wooden wall (WP1), and the unpainted wooden wall (WP2)

Insecticide	Substrate	Days post IRS								
		1	30	60	90	120	150	180	210	240^a^
Alpha-cypermethrin SC	WP1	1.00 [0.78, 1.28]	1.00 [0.81, 1.24]	0.99 [0.73, 1.34]	0.77 [0.57, 1.05]	0.81 [0.59, 1.10]	0.49 [0.34, 0.70]	0.24 [0.15, 0.38]	–	–
	WP2	0.83 [0.64, 1.08]	0.91 [0.73, 1.14]	*0.45 [0.31, 0.66]*	0.42 [0.28, 0.61]	–	–	–	–	–
	CP1	0.98 [0.77, 1.26]	0.7 [0.57, 1.04]	*0.21 [0.13, 0.35]*	–	–	–	–	–	–
	CP2	0.97 [0.73, 1.28]	*0.51 [0.36, 0.71]*	0.50 [0.36, 0.70]	–	–	–	–	–	–
Etofenprox WP	WP1	1.00 [0.79, 1.27]	1.00 [0.73, 1.37]	0.85 [0.62, 1.15]	0.81 [0.59, 1.12]	0.57 [0.40, 0.82]	0.29 [0.19, 0.45]	–	–	–
	WP2	1.00 [0.80, 1.25]	0.96 [0.72, 1.28]	0.98 [0.73, 1.31]	0.97 [0.72, 1.30]	0.86 [0.64, 1.16]	0.68 [0.49, 0.94]	0.34 [0.23, 0.50]	–	–
	CP1	1.00 [0.79, 1.26]	0.92 [0.70, 1.22]	0.86 [0.64, 1.17]	*0.40 [0.27, 0.59]*	0.52 [0.36, 0.73]	–	–	–	–
	CP2	0.97 [0.77, 1.23]	0.66 [0.48, 0.91]	0.53 [0.37, 0.77]	–	–	–	–	–	–
Lambda-cyhalothrin WP	WP1	0.99 [0.79, 1.25]	0.98 [0.72, 1.32]	0.65 [0.46, 0.91]	0.42 [0.29, 0.62]	–	–	–	–	–
	WP2	0.96 [0.77, 1.19]	0.96 [0.72, 1.28]	0.72 [0.53, 0.98]	0.62 [0.44, 0.87]	–	–	–	–	–
	CP1	0.88 [0.70, 1.11]	0.78 [0.57, 1.05]	*0.02 [0.01, 0.09]*	–	–	–	–	–	–
	CP2	0.98 [0.77, 1.24]	0.73 [0.53, 1.00]	0.58 [0.39, 0.86]	–	–	–	–	–	–
Deltamethrin WG	WP1	1.00 [0.74, 1.36]	1.00 [0.77, 1.30]	0.97 [0.75, 1.26]	0.97 [0.74, 1.27]	0.95 [0.75, 1.21]	0.94 [0.74, 1.19]	0.84 [0.64, 1.11]	0.82 [0.61, 1.10]	0.83 [0.65, 1.06]
	WP2	1.00 [0.74, 1.35]	0.97 [0.76, 1.26]	0.59 [0.45, 0.78]	0.94 [0.70, 1.25]	0.94 [0.73, 1.20]	0.93 [0.73, 1.19]	0.92 [0.70, 1.20]	0.97 [0.75, 1.26]	0.99 [0.79, 1.25]
	CP1	1.00 [0.74, 1.35]	1.00 [0.77, 1.30]	0.90 [0.70, 1.17]	0.87 [0.66, 1.15]	0.72 [0.56, 0.94]	0.68 [0.52, 0.88]	–	–	–
	CP2	1.00 [0.75, 1.33]	0.98 [0.77, 1.26]	0.84 [0.65, 1.09]	0.72 [0.53, 0.98]	0.66 [0.50, 0.88]	–	–	–	–
Bendiocarb WP	WP1	1.00 [0.78, 1.27]	1.00 [0.78, 1.28]	1.00 [0.81, 1.24]	1.00 [0.76, 1.32]	*0.53 [0.40, 0.69]*	0.61 [0.46, 0.80]	–	–	–
	WP2	1.00 [0.77, 1.29]	1.00 [0.76, 1.31]	1.00 [0.79, 1.27]	0.99 [0.76, 1.29]	*0.47 [0.36, 0.63]*	0.66 [0.51, 0.87]	–	–	–
	CP1	1.00 [0.77, 1.31]	*0.11 [0.06, 0.19]*	0.02 [0.01, 0.07]	–	–	–	–	–	–
	CP2	1.00 [0.76, 1.31]	*0.05 [0.02, 0.11]*	0.05 [0.02, 0.11]	–	–	–	–	–	–
Pirimiphos-Methyl CS	WP1	1.00 [0.78, 1.28]	1.00 [0.76, 1.32]	1.00 [0.80, 1.25]	0.98 [0.78, 1.24]	1.00 [0.79, 1.26]	1.00 [0.78, 1.28]	0.99 [0.78, 1.26]	0.97 [0.75, 1.26]	0.78 [0.62, 0.98]
	WP2	1.00 [0.78, 1.28]	1.00 [0.76, 1.32]	0.94 [0.75, 1.17]	0.79 [0.62, 1.02]	0.84 [0.65, 1.08]	0.87 [0.68, 1.12]	0.74 [0.57, 0.96]	0.65 [0.49, 0.86]	–
	CP1	1.00 [0.78, 1.27]	0.97 [0.76, 1.24]	0.99 [0.79, 1.24]	0.91 [0.72, 1.16]	0.70 [0.51, 0.95]	0.95 [0.74, 1.22]	0.64 [0.49, 0.84]	0.99 [0.77, 1.28]	0.90 [0.70, 1.16]
	CP2	1.00 [0.78, 1.29]	0.97 [0.75, 1.25]	1.00 [0.80, 1.25]	0.98 [0.77, 1.24]	1.00 [0.78, 1.28]	0.98 [0.77, 1.24]	0.97 [0.77, 1.23]	0.92 [0.72, 1.17]	0.98 [0.76, 1.25]

Italic entries indicate a statistical significance at 5% in the group comparison against the preceding month

^a^The experiments were maintained until a mortality rate of less than 80% was observed for two consecutive months

There was no significant difference between relative humidity (t = 1.453, df = 352.52, p-value = 0.15) and number of rainy days (W = 26,714, p-value = 0.72) when comparing both experimental phases, but there was a significant difference when comparing mean temperature (t = − 6.4421, df = 358.78, p-value < 0.001) (Additional file 1).

### Findings in the first phase

In the bioassays performed with the pyrethroids, the results of the tests after 24 h of spraying revealed a mortality rate above 80% for all of the surfaces used, with results reaching 100% for alpha-cypermethrin SC in WP1 and for etofenprox WP in WP1 and WP2, attesting to the efficacy of the spraying. These insecticides showed high residual activity (mortality ≥ 80%) for 4 months after spraying on at least one of the surfaces (Table 1). There were no significant relationships between the surface type, cone height, and their interaction for *An. marajoara* mortality for alpha-cypermethrin SC, etofenprox WP, and lambda-cyhalothrin WP.

#### Alpha-cypermethrin SC

**Fig. 4:**
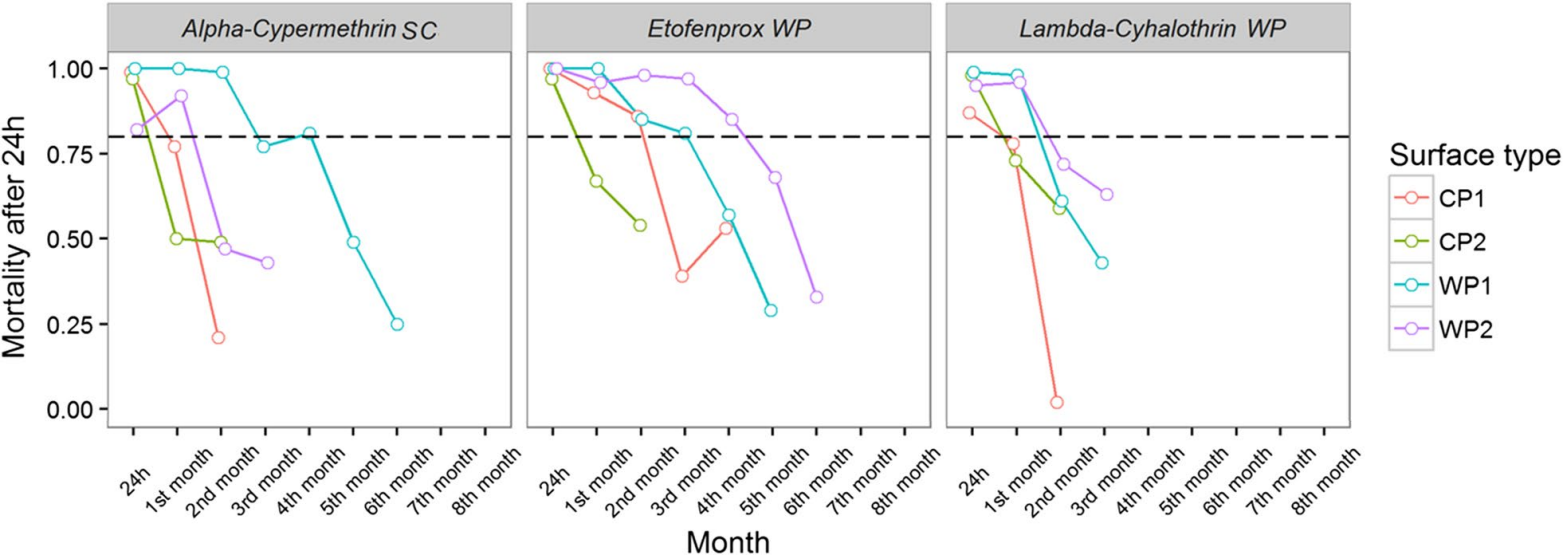
The residual effect represented by the mortality percentage for the insecticides etofenprox WP, alpha-cypermethrin SC, and lambda-cyhalothrin WP on the surfaces of painted wood (WP1), unpainted wood (WP2), plastered cement (CP1), and unplastered cement (CP2), observed after 24 h post-IRS for a period of 6 months in a field simulation trial “test house”—Macapá/AP

Overall, alpha-cypermethrin was more efficient on wooden surfaces than on cement surfaces (Fig. 4). The model results and comparisons showed a diverse pattern. CP1, CP2, and WP2 had mortality rates declining below 80% before reaching the second month of the experiment (Table 2). WP1 was the best surface for this formulation, with mortality rates above 80% for the first 4 months of the experiment (Table 1).

#### Etofenprox WP

Overall, the mortality rates declined faster on the cement walls than on the wooden ones (Fig. 4). On CP1, the mortality rate stayed above 80% for the first 2 months, while on the unplastered wall the mortality rate dropped to below 80% in the first month of the experiment. The painted wooden wall maintained high mortality rates for the first 3 months. On the unpainted wall, the mortality rate declined to below 80% in the sixth month (Tables 1, 2).

#### Lambda-cyhalothrin WP

Mortality rates for this formulation declined faster for the cement walls than for the wooden ones (Fig. 4). The mortality declined to levels below 80% on CP1 in the second month, whereas for the painted wooden walls, the mortality rates declined to levels below 80% in the 3rd month (Tables 1, 2).

### Findings in the second phase

In the bioassays performed with deltamethrin WG (pyrethroid), bendiocarb WP (carbamate), and pyrimiphos-methyl CS (organophosphate), the results of the tests after 24 h of spraying showed 100% mortality on all the surfaces used, namely WP1, WP2, CP1, and CP2, which confirmed the efficacy of the spraying (Tables 1, 2). Significant relationships were found between the surface type, cone height, and their interaction for *An. marajoara* mortality only for deltamethrin WG (OR for 1.5-m height on MCP surface: 1.508; 95% CI 1.064, 2.138).

#### Deltamethrin WG

**Fig. 5:**
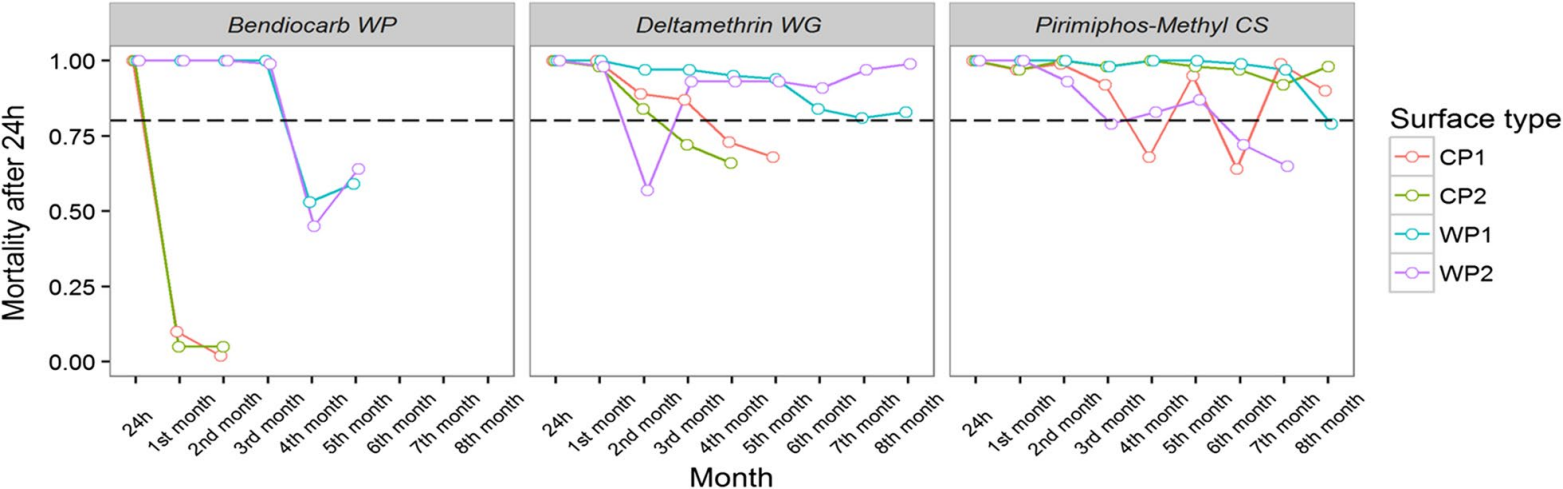
The residual effect represented by the mortality percentage for the insecticides bendiocarb WP, deltamethrin WG, and pirimiphos-methyl CS on the surfaces of painted wood (WP1), unpainted wood (WP2), plastered cement (CP1), and unplastered cement (CP2), observed after 24 h post-IRS for a period of 8 months in a field simulation trial “test house”—Macapá/AP

The mortality rates for this formulation were better on wooden surfaces than on cement ones, although the WP2 wall had a significant decrease in mortality in the second month (Fig. 5, Table 2). CP1 had a mortality rate above 80% up to the fifth month, while CP2 had a mortality rate above 80% up to the third month. For both the wooden walls, the mortality rates were maintained above 80% for all 8 months of the experiment (Tables 1, 2).

#### Bendiocarb WP

Showed better residual bioefficacy on the wooden surfaces than on cement surfaces (Fig. 5). This lasted up to 5 months on both painted and unpainted wooden surfaces. In contrast, it lasted only 1 month on the cement surfaces (Table 2), showing a rapid decline in the second month postspraying, with a mortality rate ranging between 11 and 5% on CP1 and CP2, respectively.

#### Pirimiphos-methyl CS

Overall, this formulation maintained the mortality rates above 80% on all the surfaces tested for at least 6 months (Fig. 5). On the cement surfaces and on WP1, the mortality rates were higher than 80% for all 8 months of the experiment. On WP2, the mortality declined in the seventh month of the experiment (Tables 1, 2).

## Discussion

The present study demonstrated a wide variation in the residual efficacy of six IRS products from three classes of insecticides in a field simulation (“test house”), applied to four surfaces. The type of sprayed surface and the formulation of the insecticides showed different residual results when compared with those recommended by WHOPES.

IRS can be optimized by adjusting the insecticide formulation depending on the sprayed surface [1]. Wettable powder and water dispersible granule formulations such as etofenprox WP, bendiocarb WP and deltamethrin WG have been shown to have higher residual effects on wood surfaces. The concentrated suspension formulation of alpha-cypermethrin SC was effective on painted wood, while the capsulated suspension of pirimiphos-methyl CS was effective on the wood and masonry surfaces. A mortality rate of 80% or more is the criterion adopted by the WHO to establish the residual effect of insecticides. In this study, in this study, products with residuals of 2 to 6 months were used; the efficacy results of the six insecticides showed that deltamethrin WG and pirimiphos-methyl CS met the WHO recommendation, presenting mortality rates within the established range for up to 240 days [42].

For the other insecticides, the mortality rate did not reach the minimum period of residuality for all the evaluated substrates. The complexity of monitoring the IRS vector control in field situations [51] can be overcome with information obtained from the bioassays performed in field-simulating conditions. In this study the alpha-cypermethrin concentrated suspension formulation (SC) performed better on the wood surfaces than on cement ones; however, the only surface that reached the minimum period with residual efficacy within the WHO parameters for up to 120 days (4 months) was WP1. A similar result was found in the Democratic Republic of Sao Tome and Principe, suggesting that IRS should be applied in three cycles per year [58]. For the WG-SB and WP formulations of alpha-cypermethrin on the clay and cement surfaces, the residual efficacy ranged from 11 to 16 weeks [59]. The data compiled by Dengela et al. [39] about the residual efficacy in African countries showed satisfactory performance of alpha-cypermethrin WP, varying from 4 to 10 months, on surfaces of mud, wood, cement, and other materials in the sprayed dwellings.

The residual efficacy of the etofenprox and lambda-cyhalothrin pyrethroid formulations, according to the WHOPES guidelines, ranged from 3 to 6 months. In Brazil, the recommendation for the IRS with the formulation etofenprox WP was established with a 4-month interval [24], based on the laboratory bioassays performed with WP2 panels. The results in the field simulations presented the same residual efficacy for this surface; however, Santos et al. [16] using this insecticide in the field conditions, observed residuality of up to 3 months for wooden and CP2 surfaces, but on the CP1 surface the effectiveness was lower, supporting the findings of the study. The formulation of lambda-cyhalothrin WP demonstrated a short residual efficacy on the cement and wood surfaces under evaluation in Brazil [16], being in agreement with the results obtained in this study, however studies in African countries showed satisfactory residuality, according to the period recommended by WHOPES on surfaces of cement [60] and wood [61]. Variations in the results were also described for the concentrated suspension formulation of lambda-cyhalothrin CS concerning its effectiveness on the cement surfaces [62].

The residual effect of deltamethrin varied among the different surfaces. With the WP formulation, the results displayed the expected residual efficacy (3 to 6 months) [42, 60, 63]; they surpassed the predicted period when the SC-PE formulation was used [29], and the WG formulation presented results that were below the established [52, 64, 65]. In this study, among the evaluated pyrethroids, deltamethrin WG showed the best performance, demonstrating efficacy for 8 months on the wood surfaces and maintaining a residual effect with mortality ≥ 80% for a period equal to or greater than 90 days on three of the tested surfaces. Similar results were found in previous studies [66, 67].

It was highlighted that some factors affect the residual efficacy and persistence of insecticides; for example, the activity of pyrethroids can be compromised by the rapid degradation on porous surfaces with a high absorption [16, 66]. The low residual bioefficacy of the pyrethroids on the cement surfaces compared with the wooden ones found in the present study confirms previous observations but diverges from the findings of Dunford et al. [64].

The continuous use of pyrethroids has led to an increase in the population of resistant mosquitoes. Few studies have been performed on neotropical *Anopheles* [68, 69] and although there is a shortage of records in Brazil [17, 70], this is a reality in African countries [62, 71–73], supporting the importance of using substitute products in IRS rotation schemes [1, 23]. Bendiocarb is an insecticide of the class of carbamates recommended by the WHO, with residual efficacy varying from 2 to 6 months [42]. The WP formulation achieved the expected residual efficacy on different surfaces [51, 74], but some studies reflected a short residuality from 2 to 3 months [51, 75–78].

These results are similar to those found in this study on the wooden surfaces, but on the masonry surfaces, the effectiveness was less than 30 days. The short residuality of bendiocarb compromises its use as an alternative to replace the pyrethroids in the rotation scheme for malaria control in the Brazilian Amazon since it demands a higher number of spray cycles, not presenting a cost-effective benefit for protection in endemic areas.

In the search for long-lasting insecticides, the microencapsulation technology of pirimiphos-methyl has brought significant benefits in the current context of resistance to pyrethroids. Thus, the CS encapsulation suspension formulation minimizes the limitation of the low residuality found in WP and EC emulsified concentrate formulations [42, 79], prolonging its persistence for up to 10 months on cement surfaces and for 6 to 8 months on the other surface types. The observed residual efficacy is beneficial in areas where there are up to two transmission periods per year [51, 53, 62, 67, 80, 81], corroborating the findings that residual efficacy was 8 months for the masonry and WP1 surfaces and 6 months for the WP2. The pirimiphos-methyl CS presented little variation and good performance among the tested surfaces. However, the bioassay was interrupted before the mortality rate dropped to less than 80% for two consecutive months due to the strong odour, as also reported elsewhere [27, 80].

In areas where there are no records of resistance to pyrethroids, its use should thus be considered with caution, to avoid rejection of its use by human inhabitants and inconsistent control activities (Additional file 2).

## Conclusions

The results of this study showed a variation of the residual effects of insecticides on the different tested surfaces. The residuality performance consistent with the WHO guidelines, among the pyrethroids evaluated, was observed for the insecticides deltamethrin WG for WP1, WP2 (240 days), and CP1 (90 days) surfaces and etofenprox WP for both the wood surfaces (90 and 120 days). Within this context, these insecticides would be effective in vector control programmes if applied in quarterly spray cycles. The pyrethroids alpha-cypermethrin SC and lambda-cyhalothrin WP, as well as the carbamate-bendiocarb WP, presented a lower residuality than the other insecticides studied, with a short or no period of effectiveness on the applied surfaces. On the other hand, the organophosphate pirimiphos-methyl CS was shown to be efficient on all of the surfaces so that it could be used in cycles with an interval of up to 6 months. Nevertheless, this insecticide exudes a strong odour and high toxicity in vertebrates; therefore, in countries in which resistance to pyrethroids has not been confirmed, its judicious use is recommended.

This study reveals that the variation in effects among different surfaces and the short residual effect compromises the use of insecticides, to the detriment of the cost–benefit. The persistence of the product is essential, and it should remain effective on the applied surface for sufficient time to cover the malaria transmission period. Against this background, the use of the “test house” for the field simulation is beneficial to evaluate the residual period of insecticides in order to obtain results that are more reflective of those in residences in endemic areas.

This study also recommends the performance of comparative wall bioassays using laboratory panels, field simulations, and directly in the field. The data generated from such studies can serve as an important guide to malaria control programmes, by selecting insecticides for IRS in these environments.

## Supplementary information

**Additional file 1. Figure S1:** Climate data for each experiment. Phase 1 occurred from October 2014 to April 2015 and phase 2 from May 2015 to March 2016.

**Additional file 2. Table S1:** Estimated effects of surface type, cone height and their interaction on Anopheles marajoara mortality for six different insecticide formulations. Bold entries indicate statistical significance (p < 0.05).

## Data Availability

The datasets used and/or analysed during the current study are available from the corresponding author on a reasonable request.

## Abbreviations

CP1: plastered cement
CP2: unplastered cement
CS: capsule suspension
IEPA: Institute of Scientific and Technological Research of the State of Amapá
IRS: indoor residual spraying
LLIN: long-lasting insecticidal net
NMCP: National Malaria Control Program
PAHO: Pan-American Health Organization
SC: suspension concentrate
WG: water-dispersible granules
WHO: World Health Organization
WP1: painted wood
WP2: unpainted wood
WP: wettable powder
WHOPES: World Health Organization Pesticide Evaluation Scheme

## References

[1] WHO (2015). Global technical strategy for malaria 2016–2030.

[2] WHO (2017). World malaria report 2017.

[3] Ministério da Saúde: Sistema de informação de Vigilância Epidemiológica da Malária (SIVEP-Malária). http://portalweb04.saude.gov.br/sivep_malaria/default.asp.

[4] Cardoso RF, Goldenberg P (2007). Malária no Estado do Amapá, Brasil, de 1970 a 2003: trajetória e controle. Cad Saúde Pública..

[5] Oliveira-Ferreira J, Lacerda MV, Brasil P, Ladislau JL, Tauil PL, Daniel-Ribeiro CT (2010). Malaria in Brazil: an overview. Malar J..

[6] Tauil PL, Lima JTF (1992). Critical analysis of malaria control measures in Brazil. Mem Inst Oswaldo Cruz.

[7] WHO (2013). Indoor residual spraying: an operational manual for indoor residual spraying (IRS) for malaria transmission control and elimination.

[8] WHO (2013). Recommendations for achieving universal coverage with long-lasting insecticidal nets in malaria control.

[9] Mabaso ML, Sharp B, Lengeler C (2004). Historical review of malarial control in southern African with emphasis on the use of indoor residual house-spraying. Trop Med Int Health..

[10] Tukei BB, Beke A, Lamadrid-Figueroa H (2017). Assessing the effect of indoor residual spraying (IRS) on malaria morbidity in Northern Uganda: a before and after study. Malar J..

[11] Murphy C, Ringheim K, Woldehanna S, Volmink J (2003). Reducing malarias burden: evidence of effectiveness for decision makers.

[12] Horosko S, Lima JB, Brandolini MB (1997). Establishment of a free-mating colony of *Anopheles albitarsis* from Brazil. J Am Mosq Control Assoc..

[13] Lima JB, Valle D, Peixoto AA (2004). Adaptation of a South American malaria vector to laboratory colonization suggests faster-male evolution for mating ability. BMC Evol Biol.

[14] Baia-da-Silva DC, Brito-Sousa JD, Rodovalho SR, Peterka C, Moresco G, Lapouble OMM (2019). Current vector control challenges in the fight against malaria in Brazil. Rev Soc Bras Med Trop.

[15] Malcolm CA (1988). Current status of pyrethroid resistance in anophelines. Parasitol Today..

[16] Santos RLC, Fayal AS, Aguiar AEF, Vieira DBR, Póvoa MM (2007). Avaliação do efeito residual de piretroides sobre anofelinos da Amazônia brasileira. Rev Saúde Pública..

[17] Silva APB, Alves WS, Martins AJ, Tadei WP, Santos JMM (2014). Adaptação de um bioensaio simplifcado para avaliação do status de susceptibilidade em larvas de *Anopheles darlingi* e *Anopheles marajoara* ao piretroide deltametrina. BioAssay..

[18] Deane LM (1988). Malaria studies and control in Brazil. Am J Trop Med Hyg.

[19] Loiola CCP, Silva CJMd, Tauil PL (1965). Malaria control in Brazil: 1965 to 2001. Pan Am J Public Health..

[20] Flores AV, Ribeiro JN, Neves AA, Queiroz ELRD (2004). Organoclorados: um problema de saúde pública. Ambiente Sociedade..

[21] Carneiro FF, Augusto LGS, Rigotto RM, Friedrich K, Búrigo AC (2015). Dossiê ABRASCO: um alerta sobre os impactos dos agrotóxicos na saúde Rio de Janeiro: EPSJV.

[22] Morales-Rojas H, Moss RA (2002). Phosphorolytic reactivity of o-iodosylcarboxylates and related nucleophiles. Chem Rev.

[23] Braga IA, Valle D (2007). *Aedes aegypti*: vigilância, monitoramento da resistência e alternativas de controle no Brasil. Epidemiol Serv Saúde..

[24] Brasil. Uso do Etofenprox PM 20% para Borrifação Residual Intradomiciliar no controle da malária. Transmissiveis DdVdD ed. Brasilia: Ministério da Saúde; 2014.

[25] IRAC. Insecticide Resistance Action Committee (IRAC): prevention and management of insecticide resistance in vectors of public health importance, 2nd Edn. 2011.

[26] WIN. The Worldwide Insecticide Resistance Network. 2018. https://win-network.ird.fr/. Accessed 15 Jan 2018.

[27] Tangena JA, Adiamoh M, D’Alessandro U, Jarju L, Jawara M, Jeffries D (2013). Alternative treatments for indoor residual spraying for malaria control in a village with pyrethroid- and DDT-resistant vectors in the Gambia. PLoS One.

[28] Oxborough R, Kitau J, Jones R, Feston E, Matowo J, Mosha F, Rowland M (2014). Long-lasting control of *Anopheles arabiensis* by a single spray application of micro-encapsulated pirimiphos-methyl (Actellic(R) 300 CS). Malar J..

[29] Oxborough RM, Kitau J, Jones R, Mosha FW, Rowland MW (2014). Experimental hut and bioassay evaluation of the residual activity of a polymer-enhanced suspension concentrate (SC-PE) formulation of deltamethrin for IRS use in the control of *Anopheles arabiensis*. Parasit Vectors..

[30] WHO. Guidelines for testing mosquito adulticides for indoor residual spraying and treatment of mosquito nets. WHO/CDS/NTD/WHOPES/GCDPP/2006.3. Geneva: World Health Organization; 2006.

[31] WHO. Instructions for the bioassay of insecticidal deposits on wall surfaces.VBC/81.5 (WHO/VBC/81.812). Geneva: World Health Organization; 1981.

[32] London L, Coggon D, Moretto A, Westerholm P, Wilks MF, Colosio C (2010). The ethics of human volunteer studies involving experimental exposure to pesticides: unanswered dilemmas. Environ Health..

[33] Okumu FO, Chipwaza B, Madumla EP, Mbeyela E, Lingamba G, Moore J (2012). Implications of bio-efficacy and persistence of insecticides when indoor residual spraying and long-lasting insecticide nets are combined for malaria prevention. Malar J..

[34] Galardo AKR, Galardo CD. Relatório técnico sobre o Estudo da Eficácia de Redes Impregnadas com Inseticidas e do uso de Fendona^®^ em borrifações domiciliares para o controle de *Anopheles* sp. em bioensaios de campo e laboratório no estado do Amapá—Brasil. Macapá: Instituto de Pesquisas Científicas e Tecnológicas do Estado do Amapá; 2009.

[35] Muirhead-Thomson RC (1950). DDT and gammexane as residual insecticides against *Anopheles gambiae* in African houses. Trans R Soc Trop Med Hyg.

[36] Busvine J (1951). Mechanism of resistance to insecticide in houseflies. Nature.

[37] Rapley RE (1961). Notes on the construction of experimental huts. Bull World Health Organ.

[38] Haddow AJ (2009). The mosquito fauna and climate of native huts at Kisumu, Kenya. Bull Entomol Res..

[39] Okumu FO, Moore J, Mbeyela E, Sherlock M, Sangusangu R, Ligamba G (2012). A modified experimental hut design for studying responses of disease-transmitting mosquitoes to indoor interventions: the Ifakara experimental huts. PLoS ONE.

[40] IBGE Istituto Brasileiro de Geografia e Estatítica-IBGE Cidades. 2018. http://www.ibge.gov.br/estadosat. Accessed 17 Jan 2018.

[41] FAO/WHO. Manual on development and use of FAO and WHO specifications for pesticides, 1st Edn. Geneva and Rome: Food and Agriculture Organization of the United Nations; 2016.

[42] WHOPES Recommended insecticides for indoor residual spraying against malaria vectors. 2018. http://www.who.int/whopes/Insecticides_IRS_2_Mar_2015.pdf.

[43] Póvoa M, Wirtz R, Lacerda R, Miles M, Warhurst D (2001). Malaria vectors in the municipality of Serra do Navio, State of Amapá, Amazon Region. Brazil. Mem Inst Oswaldo Cruz..

[44] Galardo AKR (2010). A importância dos *Anopheles darlingi* Root, 1926 e *Anopheles marajoara* Galvão e Damasceno, 1942 na transmissâo de malária no município de Macapá/Ap—Brasil, Tese.

[45] Griffing SM, Tauil PL, Udhayakumar V, Silva-Flannery L (2015). A historical perspective on malaria control in Brazil. Memórias do Instituto Oswaldo Cruz..

[46] WHO. Guidelines for malaria vector control. Geneva: World Health Organization; 2019. 30844152

[47] Silva APB (2014). Variabilidade do domínio IIS6 do gene do canal de sódio, associada à resistência aos inseticidas piretróides, em populações de *Anopheles darlingi* e *Anopheles marajoara* da Amazônia brasileira.

[48] WHO. Pesticides and their application—for the control of vectors and pests of public health importance. WHO/CDS/NTD/WHOPES/GCDPP/2006.1, 6th Edn. Geneva: World Health Organization; 2006.

[49] WHO. Manual for indoor residual spraying. Application of residual sprays for vector control. Geneva: World Health Organization; 2007.

[50] WHO. Manual on practical entomology in malaria. Part II. Methods and techniques. Geneva: World Health Organization; 1975.

[51] Dengela D, Seyoum A, Lucas B, Johns B, George K, Belemvire A (2018). Multi-country assessment of residual bio-efficacy of insecticides used for indoor residual spraying in malaria control on different surface types: results from program monitoring in 17 PMI/USAID-supported IRS countries. Parasit Vectors..

[52] Nikpour F, Vatandoost H, Hanafi-Bojd AA, Raeisi A, Ranjbar M, Enayati AA (2017). Evaluation of deltamethrin in combination of piperonyl butoxide (PBO) against pyrethroid resistant, malaria vector, *Anopheles stephensi* in IRS implementation: an experimental semi-filed trial in Iran. J Arthropod Borne Dis..

[53] Mashauri FM, Manjurano A, Kinunghi S, Martine J, Lyimo E, Kishamawe C (2017). Indoor residual spraying with micro-encapsulated pirimiphos-methyl Actellic 300CS against malaria vectors in the Lake Victoria basin, Tanzania. PLoS One..

[54] Abbott WS (1925). A method for computing the effectiveness of the insecticides. J Econ Entomol.

[55] Team RC. R: A language and environment for statistical computing.

[56] RStudio Team. R: a language and environment for computing. Vienna: R Foundation for Statistical Computing; 2014.

[57] Lenth RV (2016). Least-squares means: the R Package lsmeans. J Stat Softw.

[58] Tseng LF, Chang WC, Ferreira MC, Wu CH, Rampão HS, Lien JC (2008). Rapid control of malaria by means of indoor residual spraying of alphacypermethrin in the Democratic Republic of São Tomé and Príncipe. Am J Trop Med Hyg.

[59] Uragayala S, Kamaraju R, Tiwari S, Ghosh SK, Valecha N (2015). Small-scale evaluation of the efficacy and residual activity of alpha-cypermethrin WG (250 g AI/kg) for indoor spraying in comparison with alpha-cypermethrin WP (50 g AI/kg) in India. Malar J..

[60] Khosravani M, Rafatpanah A, Amiri S, Zare A (2017). the field practices of lambdacyhalothrin and deltamethrin insecticides against adult mosquitoes of *Anopheles stephensi* as the main vector of malaria: residual effects. Zahedan J Res Med Sci..

[61] Mulambalah CS, Siamba DN, Ngeiywa MM, Vulule JM (2010). Evaluation of lambda-cyhalothrin persistence on different indoor surfaces in a malaria epidemic-prone area in Kenya Res J Biol Sci..

[62] Rowland M, Boko P, Odjo A, Asidi A, Akogbeto M, N’Guessan R (2013). A new long-lasting indoor residual formulation of the organophosphate insecticide pirimiphos methyl for prolonged control of pyrethroid-resistant mosquitoes: an experimental hut trial in Benin. PLoS One.

[63] Vatandoost H, Abai MR, Abbasi M, Shaeghi M, Abtahi M, Rafie F (2009). Designing of a laboratory model for evaluation of the residual effects of deltamethrin (K-othrine WP 5%) on different surfaces against malaria vector, *Anopheles stephensi* (Diptera: Culicidae). J Vector Borne Dis..

[64] Dunford JC, Estep AS, Waits CM, Richardson AG, Hoel DF, Horn K (2018). Evaluation of the long-term efficacy of K-Othrine PolyZone on three surfaces against laboratory reared *Anopheles gambiae* in semi-field conditions. Malar J..

[65] Raeisi A, Abai M, Akbarzadeh K, Nateghpour M, Sartipi M, Hassanzehi A (2010). Residual effects of deltamethrin WG 25% as a new formulation on different surfaces against *Anopheles stephensi*, in Southeastern Iran. Iran J Arthropod Borne Dis..

[66] Etang J, Nwane P, Mbida JA, Piameu M, Manga B, Souop D (2011). Variations of insecticide residual bio-efficacy on different types of walls: results from a community-based trial in south Cameroon. Malar J..

[67] Chanda E, Chanda J, Kandyata A, Phiri FN, Muzia L, Haque U (2013). Efficacy of ACTELLIC 300 CS, pirimiphos methyl, for indoor residual spraying in areas of high vector resistance to pyrethroids and carbamates in Zambia. J Med Entomol.

[68] Suarez MF, Quinones ML, Palacios JD, Carrillo A (1990). First record of DDT resistance in *Anopheles darlingi*. J Am Mosq Control Assoc..

[69] Zamora Perea E, Balta Leon R, Palomino Salcedo M, Brogdon WG, Devine GJ (2009). Adaptation and evaluation of the bottle assay for monitoring insecticide resistance in disease vector mosquitoes in the Peruvian Amazon. Malar J..

[70] Galardo AKR, Póvoa MM, Sucupira IMC, Galardo CD, Santos RLC (2015). *Anopheles darlingi* and *Anopheles marajoara* (Diptera: Culicidae) susceptibility to pyrethroids in an endemic area of the Brazilian Amazon. Rev Soc Bras Med Trop.

[71] Winkler MS, Tchicaya E, Koudou BG, Donzé J, Nsanzabana C, Müller P, Adja AM, Utzinger J (2012). Efficacy of ICON^®^ Maxx in the laboratory and against insecticide-resistant *Anopheles gambiae* in central Côte d’Ivoire. Malar J..

[72] Oxborough RM (2016). Trends in US President’s Malaria Initiative-funded indoor residual spray coverage and insecticide choice in sub-Saharan Africa (2008–2015): urgent need for affordable, long-lasting insecticides. Malar J..

[73] Wondji CS, Coleman M, Kleinschmidt I, Mzilahowa T, Irving H, Ndula M (2012). Impact of pyrethroid resistance on operational malaria control in Malawi. Proc Natl Acad Sci USA.

[74] Randriamaherijaona S, Nepomichene T, Assoukpa J, Madec Y, Boyer S (2017). Efficacy of bendiocarb used for indoor residual spraying for malaria control in Madagascar: results with local *Anopheles* species (Diptera: Culicidae) from experimental hut trials. J Med Entomol.

[75] Akogbéto MC, Padonou GG, Gbénou D, Irish S, Yadouleton A (2010). Bendiocarb, a potential alternative against pyrethroid resistant *Anopheles gambiae* in Benin, West Africa. Malar J..

[76] Thawer NG, Ngondi JM, Mugalura FE, Emmanuel I, Mwalimu CD, Morou E (2015). Use of insecticide quantification kits to investigate the quality of spraying and decay rate of bendiocarb on different wall surfaces in Kagera region, Tanzania. Parasit Vectors..

[77] Agossa FR, Aikpon R, Azondekon R, Govoetchan R, Padonnou GG, Oussou O (2014). Efficacy of various insecticides recommended for indoor residual spraying: pirimiphos methyl, potential alternative to bendiocarb for pyrethroid resistance management in Benin, West Africa. Trans R Soc Trop Med Hyg.

[78] Kirunda J, Okello-Onen J, Opiyo EA, Rwakimari JB, de Alwis R, Okia M (2017). Assessment of Ficam VC (Bendiocarb) residual activity on different wall surfaces for control of *Anopheles gambiae* s.s. (Diptera: Culicidae) in Northern Uganda. J Med Entomol..

[79] Aïkpon R, Sèzonlin M, Tokponon F, Okè M, Oussou O, Oké-Agbo F (2014). Good performances but short lasting efficacy of Actellic 50 EC Indoor Residual Spraying (IRS) on malaria transmission in Benin, West Africa. Parasit Vectors..

[80] Tchicaya E, Nsanzabana C, Smith T, Donze J, de Hipsl M, Tano Y (2014). Micro-encapsulated pirimiphos-methyl shows high insecticidal efficacy and long residual activity against pyrethroid-resistant malaria vectors in central Côte d’Ivoire. Malar J..

[81] Haji K, Thawer N, Khatib B, Mcha J, Rashid A, Ali A (2015). Efficacy, persistence and vector susceptibility to pirimiphos-methyl (Actellic^®^ 300CS) insecticide for indoor residual spraying in Zanzibar. Parasit Vectors..

