# Supplementary material for: Efficacy of insecticides used in indoor residual spraying for malaria control: an experimental trial on various surfaces in a “test house”
Source: Malar J. 2019 Oct 10;18:345. doi: 10.1186/s12936-019-2969-6 (PMC6785876; doi:10.1186/s12936-019-2969-6)
Supplement: Supplementary file 1 — Additional file 1. Figure S1: Climate data for each experiment. Phase 1 occurred from October 2014 to April 2015 and phase 2 from May 2015 to March 2016. [file 12936_2019_2969_MOESM1_ESM.docx]

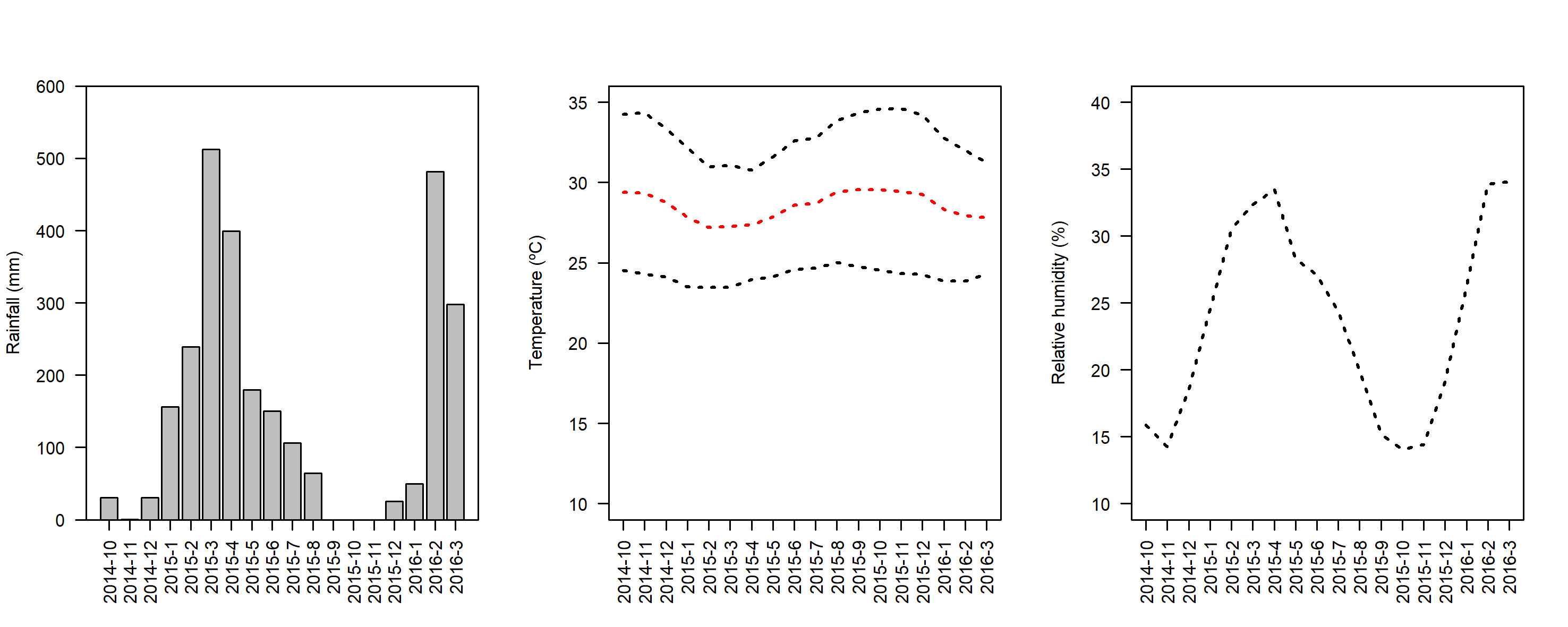


Figure S1. Climate data for each experiment. Phase 1 occurred from October 2014 to April 2015 and phase 2 from May 2015 to March 2016.
