# Supplementary material for: Efficacy of insecticides used in indoor residual spraying for malaria control: an experimental trial on various surfaces in a “test house”
Source: Malar J. 2019 Oct 10;18:345. doi: 10.1186/s12936-019-2969-6 (PMC6785876; doi:10.1186/s12936-019-2969-6)
Supplement: Supplementary file 2 — Additional file 2. Table S1: Estimated effects of surface type, cone height and their interaction on Anopheles marajoara mortality for six different insecticide formulations. Bold entries indicate statistical significance (p < 0.05). [file 12936_2019_2969_MOESM2_ESM.docx]

Table S1. Estimated effects of surface type, cone height and their interaction on *An. marajoara* mortality for six different insecticide formulations. Bold entries indicate statistical significance (p < 0.05).

| **Estimates** | **Estimate** | **Std. Error** | **95% CI** | | **Estimates** | **Estimate** | **Std. Error** | **95% CI** | |
| --- | --- | --- | --- | --- | --- | --- | --- | --- | --- |
|  |  |  | **L. bound** | **U. bound** |  |  |  | **L. bound** | **U. bound** |
| **Alphacypermethrin SC** | | | | | **Etofenprox WP** | | | | |
| **Intercept** | **0.698** | **0.151** | **0.517** | **0.938** | **Intercept** | **0.729** | **0.114** | **0.582** | **0.912** |
| Surface:ASR | 0.857 | 0.225 | 0.55 | 1.33 | Surface:ASR | 0.954 | 0.192 | 0.654 | 1.39 |
| Surface:MCP | 1.066 | 0.179 | 0.751 | 1.518 | Surface:MCP | 1.028 | 0.161 | 0.749 | 1.412 |
| Surface:MSP | 1.065 | 0.195 | 0.727 | 1.563 | Surface:MSP | 1.055 | 0.152 | 0.784 | 1.422 |
| Height:1m | 0.993 | 0.217 | 0.648 | 1.52 | Height:1m | 1.026 | 0.168 | 0.738 | 1.426 |
| Height:1.5 | 1.014 | 0.214 | 0.666 | 1.542 | Height:1.5 | 1.147 | 0.16 | 0.839 | 1.569 |
| Surface:ASR x Height:1m | 1.282 | 0.308 | 0.701 | 2.35 | Surface:ASR x Height:1m | 1.171 | 0.271 | 0.689 | 1.992 |
| Surface:MCP x Height:1m | 1.166 | 0.255 | 0.708 | 1.923 | Surface:MCP x Height:1m | 0.996 | 0.23 | 0.634 | 1.565 |
| Surface:MSP x Height:1m | 0.894 | 0.282 | 0.515 | 1.552 | Surface:MSP x Height:1m | 1.025 | 0.218 | 0.668 | 1.572 |
| Surface:ASR x Height:1m | 0.994 | 0.319 | 0.531 | 1.858 | Surface:ASR x Height:1m | 0.96 | 0.261 | 0.576 | 1.601 |
| Surface:MCP x Height:1.5m | 1.005 | 0.254 | 0.611 | 1.653 | Surface:MCP x Height:1.5m | 0.934 | 0.224 | 0.602 | 1.448 |
| Surface:MSP x Height:1.5m | 0.967 | 0.275 | 0.564 | 1.656 | Surface:MSP x Height:1.5m | 1.044 | 0.21 | 0.691 | 1.576 |
| **Lambda cyhalothrin WP** | | | | | **Bendiocarb WP** | | | | |
| **Intercept** | **0.535** | **0.159** | **0.39** | **0.727** | **Intercept** | **0.388** | **0.172** | **0.275** | **0.54** |
| Surface:ASR | 1.383 | 0.221 | 0.898 | 2.135 | Surface:ASR | 0.843 | 0.256 | 0.509 | 1.39 |
| **Surface:MCP** | **1.645** | **0.201** | **1.113** | **2.445** | **Surface:MCP** | **1.837** | **0.198** | **1.253** | **2.727** |
| **Surface:MSP** | **1.553** | **0.203** | **1.045** | **2.32** | **Surface:MSP** | **1.993** | **0.197** | **1.362** | **2.952** |
| Height:1m | 1.183 | 0.221 | 0.767 | 1.827 | Height:1m | 0.88 | 0.251 | 0.536 | 1.439 |
| Height:1.5 | 1.318 | 0.216 | 0.864 | 2.018 | Height:1.5 | 0.747 | 0.252 | 0.454 | 1.222 |
| Surface:ASR x Height:1m | 1.005 | 0.304 | 0.554 | 1.822 | Surface:ASR x Height:1m | 1.189 | 0.368 | 0.578 | 2.448 |
| Surface:MCP x Height:1m | 0.737 | 0.283 | 0.423 | 1.283 | Surface:MCP x Height:1m | 1.541 | 0.283 | 0.886 | 2.689 |
| Surface:MSP x Height:1m | 0.794 | 0.282 | 0.456 | 1.381 | Surface:MSP x Height:1m | 1.346 | 0.284 | 0.772 | 2.353 |
| Surface:ASR x Height:1m | 0.834 | 0.308 | 0.456 | 1.524 | Surface:ASR x Height:1m | 1.331 | 0.362 | 0.656 | 2.714 |
| Surface:MCP x Height:1.5m | 0.617 | 0.285 | 0.352 | 1.077 | Surface:MCP x Height:1.5m | 1.576 | 0.285 | 0.903 | 2.76 |
| Surface:MSP x Height:1.5m | 0.824 | 0.277 | 0.478 | 1.417 | Surface:MSP x Height:1.5m | 1.405 | 0.285 | 0.804 | 2.466 |
| **Deltamethrin WG** | | | | | **Pirimiphos-Methyl CS** | | | | |
| Intercept | 0.944 | 0.094 | 0.786 | 1.135 | **Intercept** | **0.788** | **0.077** | **0.677** | **0.916** |
| Surface:ASR | 0.864 | 0.143 | 0.652 | 1.143 | Surface:ASR | 1.23 | 0.105 | 1.001 | 1.513 |
| Surface:MCP | 0.923 | 0.123 | 0.725 | 1.174 | Surface:MCP | 1.136 | 0.106 | 0.923 | 1.399 |
| Surface:MSP | 0.956 | 0.122 | 0.752 | 1.216 | Surface:MSP | 0.939 | 0.115 | 0.75 | 1.176 |
| Height:1m | 0.934 | 0.133 | 0.719 | 1.213 | Height:1m | 1.199 | 0.107 | 0.973 | 1.478 |
| **Height:1.5** | **0.748** | **0.139** | **0.569** | **0.982** | Height:1.5 | 1.228 | 0.105 | 1 | 1.509 |
| Surface:ASR x Height:1m | 1.169 | 0.203 | 0.785 | 1.74 | Surface:ASR x Height:1m | 0.841 | 0.147 | 0.631 | 1.122 |
| Surface:MCP x Height:1m | 1.124 | 0.173 | 0.801 | 1.577 | Surface:MCP x Height:1m | 0.925 | 0.147 | 0.694 | 1.234 |
| Surface:MSP x Height:1m | 1.045 | 0.174 | 0.743 | 1.471 | Surface:MSP x Height:1m | 0.951 | 0.158 | 0.698 | 1.296 |
| Surface:ASR x Height:1m | 1.341 | 0.206 | 0.896 | 2.008 | Surface:ASR x Height:1m | 0.825 | 0.146 | 0.62 | 1.098 |
| **Surface:MCP x Height:1.5m** | **1.508** | **0.178** | **1.064** | **2.138** | Surface:MCP x Height:1.5m | 0.91 | 0.146 | 0.683 | 1.211 |
| Surface:MSP x Height:1.5m | 1.403 | 0.176 | 0.994 | 1.982 | Surface:MSP x Height:1.5m | 1.058 | 0.155 | 0.781 | 1.432 |
